# Dinutuximab Beta for the Treatment of High-Risk Neuroblastoma: Data from the Hungarian Pediatric Oncology Network

**DOI:** 10.3390/jcm14186641

**Published:** 2025-09-20

**Authors:** Márk Hernádfői, Márton Szabados, Edit Brückner, Ágnes Varga, Péter Hauser, Gábor Ottóffy, Ágnes Vojcek, Krisztina Csanádi, Gabriella Kertész, Zsuzsanna Jakab, Gergely Agócs, Miklós Garami

**Affiliations:** 1 Centre for Translational Medicine, Semmelweis University, 1085 Budapest, Hungary; hernadfoi.mark@bethesda.hu (M.H.); szabados.marton@semmelweis.hu (M.S.); agocs.gergely@semmelweis.hu (G.A.); 2 Bethesda Children’s Hospital, 1146 Budapest, Hungary; 3 Pediatric Center, Semmelweis University, 1094 Budapest, Hungary; bruckner.edit@semmelweis.hu (E.B.); hauserpeti@yahoo.com (P.H.); dr.jakab.zsuzsa@gmail.com (Z.J.); 4 Velkey László Children’s Health Center, B.A.Z. County Central Hospital and University Teaching Hospital, 3529 Miskolc, Hungary; agnesvargadr@gmail.com; 5 Department of Pediatric Bone Marrow & Stem Cell Transplant, South-Pest Hospital Centre–National Institute for Infectology and Hematology, 1097 Budapest, Hungary; kerteszgabi77@gmail.com; 6 Faculty of Health Sciences, University of Miskolc, Miskolc-Egyetemváros, 3515 Miskolc, Hungary; 7 Department of Pediatrics, University of Pécs Medical School, 7623 Pécs, Hungary; ottoffy.gabor@pte.hu (G.O.); vojcek.agnes@pte.hu (Á.V.); 8 Heim Pál National Pediatric Institute, 1089 Budapest, Hungary; csakri.dr@gmail.com; 9 Department of Biophysics and Radiation Biology, Semmelweis University, 1094 Budapest, Hungary

**Keywords:** anti-GD2 monoclonal antibody, dinutuximab beta, high-risk neuroblastoma, immunotherapy, maintenance therapy, refractory and relapsed neuroblastoma

## Abstract

**Background/Objectives:** The anti-GD2 monoclonal antibody dinutuximab beta has become standard of care maintenance therapy for high-risk neuroblastoma (HR-NB) in the first-line setting and is also approved in the relapsed/refractory setting. We present a retrospective review of 37 children with HR-NB included in the Hungarian Childhood Cancer Registry who received dinutuximab beta (first-line maintenance therapy, n = 31; relapsed/refractory, n = 6). **Methods:** All patients received dinutuximab beta continuously over the first 10 days of each 35-day cycle, with dosing based on body surface area/weight. Five cycles were planned, with further cycles administered at the treating physician’s discretion. **Results:** At data cutoff, the overall disease control rate was 54.1% (20/37) (complete response, 51.4% (19/37); partial response, 0.0% (0/37), stable disease, 2.7% [1/37]); two patients (5.4%) had progressive disease, and 15 patients (40.5%) had died. The 5-year overall survival (OS) and event-free survival (EFS) rates in the overall population were 63.3% (95% confidence interval, 49.1−81.7) and 56.2% (95% confidence interval, 42.1−75.0), respectively. Grade 3 or 4 adverse events (including blood and lymphatic system disorders, hypoxia, hypotension, and capillary leak syndrome) were generally consistent with dinutuximab beta’s known safety profile. **Conclusions:** Dinutuximab beta was an effective immunotherapy for patients with HR-NB in routine clinical practice, with a generally manageable side effect profile.

## 1. Introduction

The incidence of neuroblastoma, a malignancy of the peripheral sympathetic nervous system [1], is approximately 3–15 cases per million children (0–14 years of age) worldwide [2], making it the most commonly diagnosed cancer during infancy and the most common extracranial solid tumor in children [3]. The median age of patients at diagnosis is 17 months and 36% of patients are diagnosed before their first birthday [4]. In 2006, the Children’s Oncology Group (COG) updated the age cutoff for risk stratification in neuroblastoma from 12 to 18 months [5]. This change was based on retrospective analyses showing that toddlers–previously considered high-risk based on a 12-month cutoff–achieved equally good outcomes when treated with intermediate-risk therapy [5]. This adjustment helped ensure that patients were not overtreated while still receiving effective therapy. Approximately half of patients diagnosed with neuroblastoma have high-risk neuroblastoma (HR-NB) [6], which is associated with a poor 5-year survival rate (40–50%) [7,8]. Of those patients with HR-NB, 20% will be refractory to treatment and more than half of those who do respond to therapy will relapse [6].

For patients with HR-NB, anti-GD2 monoclonal antibodies, such as dinutuximab beta, have become the standard of care maintenance therapy in the first-line setting [9]. First-line intense multimodal therapy for patients with HR-NB typically includes induction chemotherapy, surgery, consolidation myeloablative chemotherapy followed by autologous stem cell transplantation (ASCT) and radiation therapy, as recommended by the International Society of Paediatric Oncology European Neuroblastoma (SIOPEN) group and other European protocols [10]. The results of a study undertaken under the auspices of SIOPEN demonstrated that the addition of dinutuximab beta in the maintenance setting increased 5-year overall survival (OS) to over 60% for the first time in patients with HR-NB [11], and, along with another study undertaken by SIOPEN [12], led to its approval for patients with HR-NB who are ≥12 months of age and who have achieved at least a partial response (PR) to induction chemotherapy, and received myeloablative chemotherapy and stem cell transplant [13]. SIOPEN recommends dinutuximab beta administered at 10 mg/m^2^ per day for 10 days as a continuous intravenous infusion (cumulative dose: 100 mg/m^2^) as the standard of care for patients with HR-NB [12]. Dinutuximab beta was also approved for the management of patients with relapsed or refractory HR-NB with or without residual disease [13], based on the results of another study undertaken by the SIOPEN group [14]. While the SIOPEN HR-NBL1 trial described above spans 23 countries (188 sites; 3569 patients) [15], European registry/policy data show persistent east–west inequalities (e.g., higher proportion of undiagnosed patients, diagnostic delays, and variable access to novel therapies in Southern/Eastern Europe compared to Western Europe), potentially yielding a different real-word population compared to that in predominantly Western European trial settings. In addition, existing real-world studies from Eastern European countries are relatively small. Thus, data from a registry linked to the Hungarian Pediatric Oncology Network (HuPON) may help fill this regional evidence gap.

HuPON is a professional organization dedicated to advancing the care of children with cancer and to oncology research in Hungary (https://www.gyermekdaganat.hu/, accessed on 5 September 2025). HuPON was established over 50 years ago and currently comprises eight centers across Hungary. It provides centralized treatment protocols and national registration via the Hungarian Childhood Cancer Registry. Data from HuPON indicate that the age-specific incidence rate for neuroblastoma in Hungary is 12.8 per million children per year [16].

We present data from patients with HR-NB included in the Hungarian Childhood Cancer Registry who received dinutuximab beta either as first-line maintenance therapy or in the relapsed or refractory setting.

## 2. Materials and Methods

A retrospective review was undertaken of data from patients with HR-NB who had received dinutuximab beta immunotherapy first-line or in the relapsed or refractory setting in one of the following five HuPON centers in Hungary between October 2018 and February 2023: Pediatric Center, Semmelweis University, Budapest; Heim Pál National Pediatrics Institute, Budapest; Department of Pediatric Bone Marrow & Stem Cell Transplant, South-Pest Hospital Centre–National Institute for Hematology and Hematology, Budapest; Department of Pediatrics, University of Pécs, Pécs; and Velkey László Children’s Health Center, B.A.Z. County Central Hospital and University Teaching Hospital, Miskolc. Data were collected from the Hungarian Childhood Cancer Registry and from the participating centers directly.

The parents/legal guardians of the patient or the patient themselves provided informed consent for treatment.

### 2.1. Patients and Treatment

HR-NB was defined using the International Neuroblastoma Staging System (INSS) classification system, i.e., patients ≥12 months of age with INSS stage 4 neuroblastoma; patients with INSS stage 3, 4 or 4S neuroblastoma and *MYCN* amplification; and patients with INSS stage 2 neuroblastoma with *MYCN* amplification and unfavorable histology [17,18]. Patients aged 12–18 months were eligible for inclusion when they met SIOPEN high-risk criteria—i.e., stage M (metastatic) neuroblastoma diagnosed >365 days of age, irrespective of *MYCN* status, or *MYCN*-amplified disease at any stage/age. Patients with disseminated relapsed neuroblastoma were also included, irrespective of their age and disease stage at diagnosis. All patients were required to have measurable or evaluable disease at initiation of dinutuximab beta therapy; patients in complete remission at the start of dinutuximab beta treatment were not included. In addition to first-line high-risk cases, patients who commenced dinutuximab beta in the relapse setting irrespective of their diagnostic INSS stage or *MYCN* status were also included, contingent on measurable/evaluable disease at treatment start. This is consistent with the authorized use of dinutuximab beta for relapsed/refractory neuroblastoma. Patients with primary HR-NB were treated according to the HR-NB Study 1/SIOPEN protocol, as recommended by HuPON: induction therapy (rapid COJEC: cisplatin [C], vincristine [O], carboplatin [J], etoposide [E], and cyclophosphamide [C]) [19], with a maximum of 2 cycles of topotecan-vincristine-doxorubicin (TVD) in the event of an insufficient treatment response [20], followed by consolidation therapy (megachemotherapy with busulfan and melphalan followed by ASCT) [21] and maintenance therapy with dinutuximab beta [9,11]. Patients with HR-NB who had not been treated with dinutuximab beta during their initial treatment received dinutuximab beta during their relapse phase. Local radiotherapy was given after high-dose chemotherapy with ASCT and before starting dinutuximab beta maintenance, in line with SIOPEN/HuPON practice.

All patients received anti-GD2 antibody dinutuximab beta (ch14.18/CHO; DB; Recordati, Schiphol-Rijk, The Netherlands, B.V.) continuously over the first 10 days of each 35-day cycle. The daily dose of dinutuximab beta was calculated individually, depending on the body surface area/body weight of patients (Table S1). Five cycles of dinutuximab beta were planned; further cycles could be administered at the discretion of the treating physician. The first cycle was administered inpatient; subsequent cycles were delivered as home-based continuous infusions in clinically eligible patients, with daily check-ins and 24/7 on-call coverage. Home administration was restricted to families trained in pump use, with no significant prior infusion toxicities, no concomitant intravenous medications, and travel time to the treating center <30–60 min. Each patient could receive dinutuximab beta in the first-line maintenance setting and again in the relapsed setting, as necessary. None of the patients received dinutuximab beta with interleukin-2. The standard supportive treatments recommended for patients receiving dinutuximab beta via long-term infusion were given [13,22].

Intolerable toxicity was typically defined as any Grade 3 or 4 toxicity that failed to reduce to Grade 1 or 2 before the commencement of the subsequent treatment cycle, or Grade 4 hematological toxicities that persisted across treatment cycles. In cases where consolidation treatment was intended, the number of dinutuximab beta plus chemotherapy cycles depended on the treatment’s effectiveness. Treatment stopped either when the response was adequate for administering megachemotherapy or upon the diagnosis of progressive disease (PD).

### 2.2. Assessments and Outcomes

Tumor responses were evaluated using the International Neuroblastoma Response Criteria for metastatic lesions [23], at baseline, after 5 cycles of dinutuximab beta and every 2 cycles thereafter in patients who received >5 cycles, and at any time when the treating physician suspected disease progression/relapse. The overall response is determined by integrating the responses from each individual component–specifically, soft tissue, bone, and bone marrow disease. Each component is carefully evaluated to ensure its quality is sufficient for a complete overall assessment. To achieve an overall complete response (CR), every involved component must individually demonstrate a complete response. Tumors were evaluated locally by a multidisciplinary team comprising oncologists, surgeons and radiologists.

In patients with measurable disease, tumors were assessed radiographically using computed tomography and/or magnetic resonance imaging. Patients with ^123^I or ^131^I-meta-iodobenzylguanidine (MIBG)-positive tumors were evaluated for MIBG response every 2 cycles after cycle 5. Patients with MIBG non-avid tumors were evaluated using positron emission tomography. Bone marrow involvement was assessed bilaterally using routine cytomorphological examination and histopathological examination with immunostaining.

OS was defined as the time from diagnosis until death from any cause and event-free survival (EFS) was defined as the time from diagnosis until the occurrence of a disease-related event, including progression of disease.

Patients were monitored for adverse events (AEs), which were coded using the Common Terminology Criteria for Adverse Events version 5.0 (CTCAE v5.0). CTCAE v5.0 was also used to determine the severity of AEs.

### 2.3. Statistical Analysis

Data cutoff for survival analysis was 9 April 2025. OS and EFS were analyzed, stratified by relevant predictors, including *MYCN* amplification status and use of dinutuximab beta as first-line treatment. Patients were censored at the date of the last assessment. The analysis was performed using the R programming language (R version 4.4.1 [9 April 2025]) [24], specifically employing the survival (version 3.7.0) [25,26] and survminer (version 0.4.9) [27] packages. The survival::Surv() function was used to create survival objects for OS and EFS based on follow-up time and event status. Right-censored data were labeled accordingly. The Kaplan–Meier method was applied to estimate survival probabilities for OS and EFS, both for the entire cohort and for specific subgroups based on *MYCN* amplification status and dinutuximab beta treatment. The survival::survfit() function was used. The survminer::ggsurvplot() function was used to visualize survival curves with confidence interval (CI) bands, with survival probabilities presented as percentages. For visual clarity, tables showing the number of patients at risk, cumulative events, and cumulative censoring were displayed below each Kaplan–Meier curve. Median survival times were extracted from the Kaplan–Meier survival tables, along with 95% CIs for each time point. The Cox proportional hazards model was used to assess the impact on survival of the subgrouping predictors *MYCN* amplification and dinutuximab beta as first-line treatment. We used the survival::coxph() function for this purpose. No patients were lost to follow-up. Right censoring occurred only for individuals who had not reached the endpoint by the end of the study period.

Analyses were stratified by line of therapy at first dinutuximab beta exposure: first-line maintenance vs. relapsed/refractory (dinutuximab beta given after relapse/progression), following prior real-world reports. Patients remained in their subgroup for all primary analyses.

## 3. Results

### 3.1. Patients

In total, 37 patients with HR-NB received dinutuximab beta at one of five HuPON centers in Hungary between October 2018 and February 2023 (Table 1). Dinutuximab beta was received as first-line therapy by 31 (83.8%) patients, six (16.2%) patients received it for the treatment of relapsed HR-NB (four of the patients had distant metastases); a summary of outcomes for the two groups stratified by line of treatment is provided in Table S2. Patients were followed up (OS) for a maximum of 11.8 years.

At dinutuximab beta initiation, all included patients had measurable or evaluable disease by design (see eligibility). Baseline status was assessed using INRC across soft tissue, bone, and marrow components. Semi-quantitative MIBG scores (Curie/SIOPEN) were not available for this retrospective dataset and are not presented.

The majority of patients were male (n = 26, 70.3%) and the median age was 39.2 months (range 22 days–12.4 years). *MYCN* was amplified in 15 (40.5%) patients, including 11 of the 27 patients with INSS stage 4 disease and a patient with stage 2 disease and unfavorable histopathology. Three patients had been diagnosed under the age of 12 months (1 patient stage 3 at diagnosis, who received dinutuximab beta as second-line treatment; 1 patient stage 4S; and 1 patient stage 2).

Most primary tumors were found in the adrenal glands (91.9%), with two (5.4%) found in the abdomen and one (2.7%) in the lymph nodes. At diagnosis, only four (10.8%) patients did not have metastases, with the majority of patients having metastases in two or more compartments (59.5%), most commonly the bone marrow, bone and lymph nodes.

### 3.2. Treatment

Five cycles of dinutuximab beta were received by 23 (62.2%) patients (Figure S1, Table 2). Twelve (32.4%) patients were administered fewer than 5 cycles of dinutuximab beta. In addition, one (2.7%) patient received 6 cycles of dinutuximab beta in total, 5 cycles first-line and an additional cycle to treat relapsed disease (RIST [rapamycin (R) and dasatinib (S) plus chemotherapy with irinotecan (I) and temozolomide (T)] [28], 1 cycle of N5, 1 cycle of N6, and MIBG plus haploidentical hematopoietic stem cell transplant [haplo-SCT]); therapy was discontinued due to the patient’s death. One further patient (2.7%) received 9 cycles of dinutuximab beta, 5 cycles first-line (SIOPEN HR-NBL) and an additional 4 cycles to treat relapsed disease (4 cycles of N5, 4 cycles of N6, and MIBG + haplo-SCT).

In total, 11 (29.7%) patients received additional treatment after dinutuximab beta (Table 2). The treatments varied greatly in their composition and intensity, and the only regimen used by more than one patient was RIST on its own (six [16.2%] patients).

### 3.3. Tumor Response and Survival Analysis

At data cutoff, the objective response rate was 51.4% (19/37), with a CR in 19 (51.4%) patients and a PR in zero (0.0%) patients (Table 3). An additional one patient (2.7%) had SD, giving a disease control rate of 54.1% (20/37). Two (5.4%) patients had PD and 15 (40.5%) patients had died at data cutoff.

**Figure 1:**
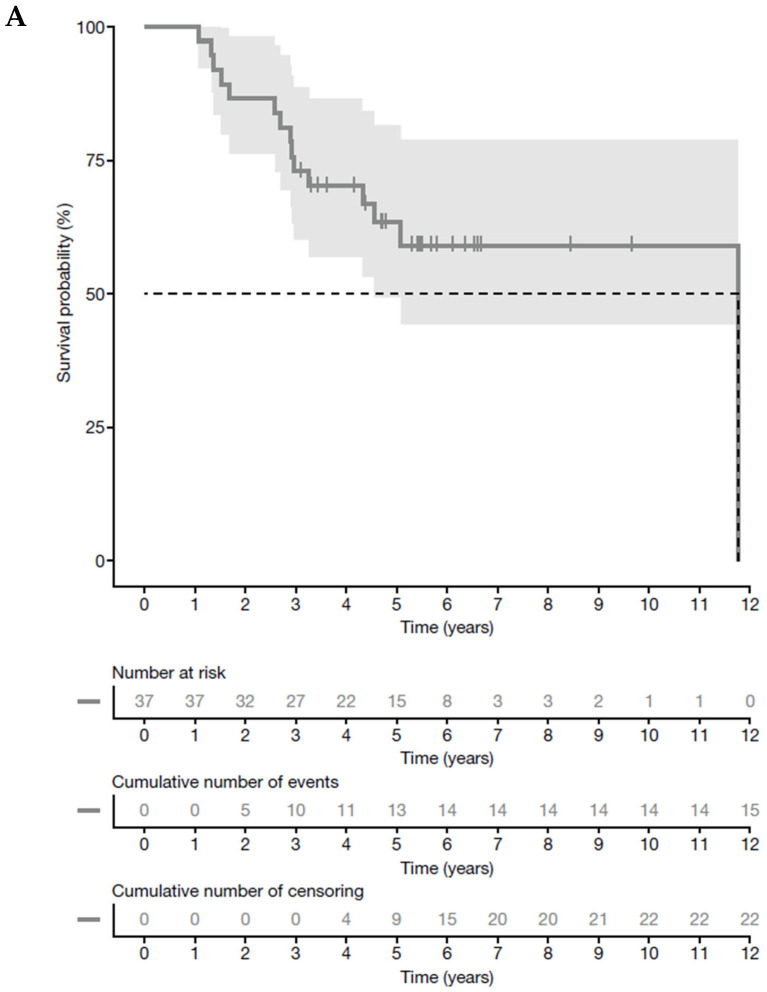

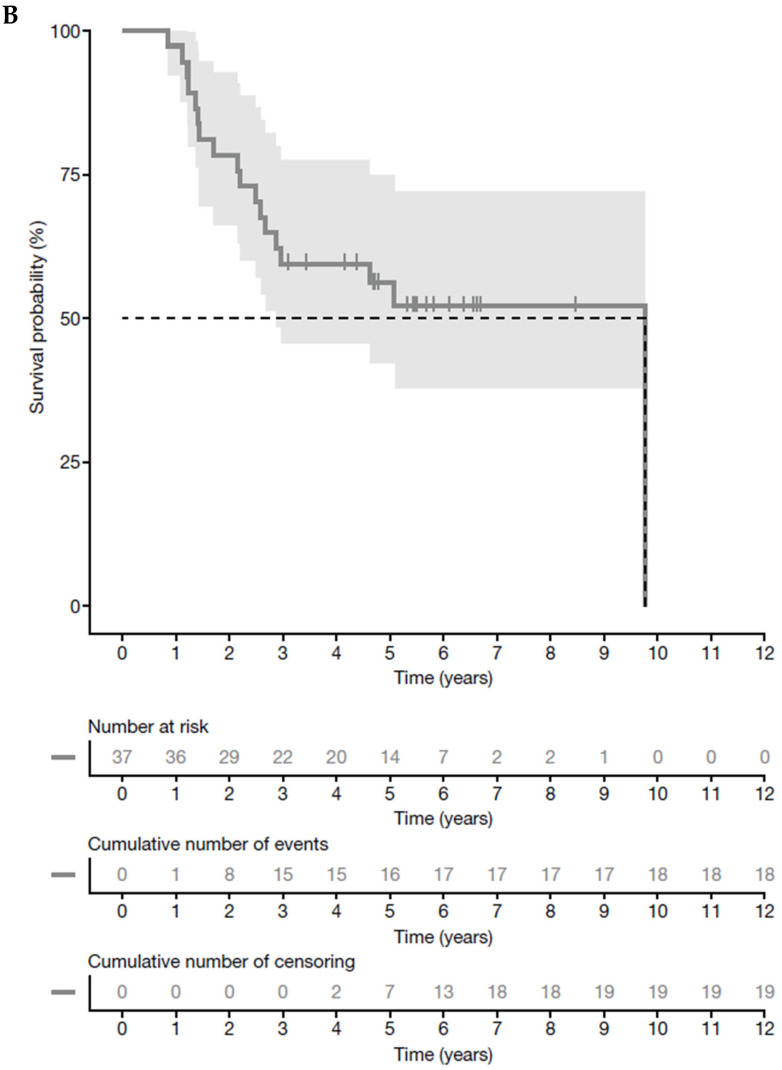
Kaplan–Meier curves for (**A**) OS and (**B**) EFS for the whole cohort. Event timelines were calculated from initial diagnosis. EFS, event-free survival; OS, overall survival.

The median OS for the entire cohort was 11.8 years (95% CI 4.6—not available [NA; the upper bound cannot be determined due to the low number of patients reaching endpoint]) and the median EFS was 9.8 years (95% CI 2.9—NA) (Figure 1). The 5-year OS and EFS rates in the overall population were 63.3% (95% CI 49.1%−81.7%) and 56.2% (95% CI 42.1%−75.0%), respectively. In the cohort treated with dinutuximab beta as first-line therapy, the median overall survival (OS) could not been determined. The event-free survival (EFS) was 5.1 years (95% CI 2.5—NA), with 5-year OS rate EFS of 59% (95% CI 43%−80%) and 55% (95% CI 40%−75%), respectively (Figure S2A,B). For the cohort receiving dinutuximab beta as second-line therapy, neither the median OS nor EFS were able to determine; the 5-year OS rate was 83% (95% CI 58%−100%), and the 5-year EFS rate was 67% (95% CI 38%−100%). The OS and EFS rates did not differ significantly between the first-line and second-line cohorts (*p* = 0.55 and *p* = 0.73, respectively). In the *MYCN* amplification negative cohort, the median OS was 11.8 years (95% CI 4.6–NA) and the median EFS was 5.1 years (95% CI 2.7–NA), with a 5-year OS rate of 67% (95% CI 48–93%) and a 5-year EFS of 56% (95% CI 38–83%). In the *MYCN* amplification positive cohort, the median OS and EFS were not reached and the 5-year OS and EFS rates were 53% (95% CI 33–86%) and 53% (95% CI 33–86%), respectively **(**Figure S2C,D). The OS and EFS rates did not differ significantly between the *MYCN* amplification negative and positive cohorts (*p* = 0.33 and *p* = 0.84, respectively).

The Cox proportional hazards model revealed no statistically significant differences in overall survival (OS) or event-free survival (EFS) based on treatment setting (first-line vs. second-line dinutuximab beta), *MYCN* amplification status (present vs. absent), or the interaction between these factors. Additionally, the models as a whole do not explain a significant amount of the variation in overall survival, possibly due to the limited sample size.

### 3.4. Toxicities

The most common Grade 3 or 4 AEs (affecting ≥5% of patients), categorized using CTCAE v5.0, were blood and lymphatic system disorders–other (37.8%), hypoxia (37.8%), hepatobiliary disorders–other (29.2%), hypotension (27.0%), capillary leak syndrome (13.5%), diarrhea (8.1%), generalized edema (5.4%) and urinary tract infection (5.4%) (Table S3). A small number of isolated grade 3/4 AEs were reported (n = 1, 2.7% each), including Epstein–Barr virus reactivation, pulmonary edema, and typhlitis. Only five (13.5%) patients experienced Grade 4 AEs (hepatobiliary disorders–other, n = 2; capillary leak syndrome, n = 1; acute respiratory distress syndrome, n = 1; typhlitis, n = 1).

## 4. Discussion

We conducted a retrospective review of data from patients with HR-NB included in the Hungarian Childhood Cancer Registry treated with dinutuximab beta either as first-line maintenance therapy (n = 30) or in the relapsed/refractory setting (n = 7). Patients were treated according to the HR-NB Study 1/SIOPEN protocol, without interleukin-2. The overall disease control rate was 54.1%, with 51.4% of patients experiencing a CR, 0.0% experiencing a PR and 2.7% with SD. Patients treated with dinutuximab beta in the first-line setting demonstrated a 5-year OS rate of 58% and a 5-year EFS rate of 56%, while corresponding values for patients who received dinutuximab beta in the relapsed/refractory were 71% and 54%, respectively.

Grade 3 or 4 AEs experienced by the patients were generally consistent with dinutuximab beta’s known safety profile [13], and included blood and lymphatic system disorders, hypoxia, hypotension, capillary leak syndrome, diarrhea, generalized edema, and urinary tract infection. Hepatobiliary disorders were experienced by 29.2% of the patients. A range of rarer Grade 3 or 4 AEs (each affecting only one patient) were also reported, including acute respiratory distress syndrome, allergic disorders, anaphylaxis, depressed level of consciousness, Epstein–Barr virus reactivation, pulmonary oedema, and typhlitis. AEs were generally manageable with supportive care and their occurrence decreased with successive treatment cycles. Only five patients experienced Grade 4 AEs. These findings highlight the importance of monitoring and managing potential AEs in patients undergoing treatment with dinutuximab beta.

All but four patients (89.2%) had metastatic disease at baseline, and the majority (59.5%) had metastases in two or more compartments [29].

Approximately one third of patients (29.7%) received additional treatment following their initial dinutuximab beta therapy, which encompassed a variety of regimens. In the first-line setting, the most commonly used treatment was RIST, which was utilized alone in six (16.2%) patients and following radiotherapy in one patient. RIST was also used in one patient following dinutuximab beta treatment in the second-line setting, together with one cycle each of N5 and N6, MIBG therapy, and haplo-SCT. RIST is a novel multimodal metronomic treatment protocol [30]. The regimen combines two molecular-targeted drugs, rapamycin (a mammalian target of rapamycin [mTOR] inhibitor) and dasatinib (a multi-kinase inhibitor and immunosuppressant), with conventional chemotherapy (irinotecan and temozolomide) [30]. Pre-treatment with rapamycin and dasatinib is believed to synchronize cell cycling and have apoptotic and chemo-sensitizing effects, while metronomic therapy is thought to decrease toxicity and thwart drug resistance, and to provide a more ‘anti-tumorigenic’ microenvironment [30,31]. Another innovative metronomic therapy, published by M. Kieran [32,33], involving celecoxib, thalidomide, fenofibrate, cyclophosphamide, and etoposide, was administered to one patient following dinutuximab beta therapy in the first-line setting. The variety of treatment approaches employed highlights the personalized nature of post-cancer therapy, tailored to the specific needs and responses of each patient. It is, however, noteworthy that further treatment after dinutuximab therapy was not pursued in 26 (70.3%) patients.

Several other real-world studies have investigated the effectiveness and tolerability of dinutuximab therapy in patients with HR-NB [34,35,36]. A retrospective review was conducted of 54 patients with HR-NB who received maintenance therapy with dinutuximab beta either first-line (n = 37) or in the relapsed or refractory setting (n = 17) at three centers in Poland [33]. Following dinutuximab beta treatment in the first-line setting, 28 (75.7%) patients had a CR, two (5.4%) had a PR, three (8.1%) had PD, and four (10.8%) relapsed [33]. Median OS was 24.4 months, and the 3-year rates of OS and progression-free survival (PFS) were 80% and 63%, respectively [33]. Nine (24.3%) patients died, eight (21.6%) due to PD and one (2.7%) due to septic complications (2.7%) [34]. Following dinutuximab beta treatment in the second-line setting, 11 (64.7%) patients had a CR, two (11.8%) had a PR, one (5.9%) had SD, and three (17.6%) had PD or had relapsed [34]. Median OS was 33.1 months, and the 3-year rates of OS and PFS were 86% and 75%, respectively [33]. Three (17.6%) patients died, two (11.8%) from PD and one (5.9%) from late complications (lung fibrosis) [33]. Dinutuximab was generally well tolerated in both settings [34]. Grade 3 toxicities were manageable and, in most cases, did not result in treatment discontinuation [33]. A retrospective chart review of patients with HR-NB treated at a single center in Croatia compared outcomes between individuals treated with dinutuximab beta (n = 11) versus those who did not receive immunotherapy (n = 12) [35]. Of the patients treated with dinutuximab beta, eight (72.7%) had a CR (median duration of response, 62 months), one (9.1%) had SD and two (18.2%) died due to PD [35]. Of those who did not receive immunotherapy, one (8.3%) had a CR and (91.7%) had 11 patients died due to PD [34]. Median EFS was higher in patients treated with dinutuximab beta than in those who did not receive immunotherapy (40.0 vs. 12.9 months), as was median OS (56.0 vs. 20.7 months) [35]. Dinutuximab beta was generally well tolerated and all AEs were manageable [34]. The most common AEs associated with dinutuximab beta therapy were fever, fluid retention indicative of capillary leak syndrome, hypotension, hypoxia, and diarrhea [35]. Finally, in a case series conducted at a single center in Bratislava, six of seven (85.7%) patients with HR-NB treated with dinutuximab beta in the first-line setting achieved a CR (median response duration, 21.5 months) and one (14.3%) had SD 21 months after completing treatment [35]. AEs were generally managed with supportive care and included mild-to-moderate pain, capillary leak syndrome, nausea/vomiting/anorexia, mild allergic reactions, irritability, hepatopathy, and hematological abnormalities [36]. The CR rate in patients treated in the first-line setting in the current study (40.5%) was lower than the rates reported in the Polish (75.7%) [33] and Bratislavan (85.7%) [36] studies, and the death rate in the current study (35.1%) was higher than the overall death rate in the Polish study (22.2% [12/54]) [33]. These differences may perhaps reflect the fact that the majority of patients in the current study were in the metastatic stage of the disease. The 5-year OS and EFS rates in patients treated in the second-line setting in the current study were surprisingly high (71% and 54%, respectively), with few individuals reaching the endpoint and a high number of censored cases. However, while the relatively small sample size (n = 6) and wide confidence intervals make these findings exploratory,, they are nevertheless consistent with the high 3-year OS and PFS rates reported for patients treated in the second-line setting in the Polish study (86% and 75%, respectively) [34]. The safety findings of the current study were generally consistent with those of these other studies.

The predominance of Tier 2M cases is a notable feature of the current study population, perhaps indicating either a need for earlier detection and treatment of neuroblastoma in Hungarian clinical practice, or a relatively cautious approach to using dinutuximab beta earlier in the first-line setting at the present time. However, although 89.2% of patients presented with metastatic disease and three cases were initially misdiagnosed, suggesting possible delays, we cannot confirm a rural diagnostic delay in Hungarian pediatric oncology clinics (historical data show no delay vs. Western Europe). Nonetheless, general practitioner workforce shortages/urban–rural performance gaps and higher childhood-cancer mortality outside Budapest indicate that health-system factors could contribute to this [16,37,38,39], while payer authorization limits and the 62.2% five-cycle completion rate for dinutuximab beta observed in our study support a concurrent ‘cautious use’ interpretation. The study’s findings might therefore influence future treatment protocols or patient management in Hungary or similar settings. The study is limited by its retrospective design, which may have resulted in selection bias, and also by the lack of a comparator group. In addition, there was heterogeneity in subsequent treatment that each patient received, making it difficult to attribute outcomes solely to dinutuximab beta. Nevertheless, its findings are generally consistent with those of other real-world studies [34,35,36]. When taken together, this evidence indicates that dinutuximab beta is an effective immunotherapy for patients with HR-NB in routine clinical practice when used either first-line or in the relapsed or refractory setting, with a generally manageable side effect profile. Our study, which is based on data from HuPON, complements prior European real-world evidence that was multi-institutional but limited to three Polish centers, or single-center series from Croatia and Slovakia, thus providing population-level Hungarian data in a unified national framework. Our findings also support the rationale for future prospective trials in patients with HR-NB to further clarify the efficacy and safety of dinutuximab beta in different patient subgroups and settings.

## 5. Conclusions

This retrospective study demonstrates that dinutuximab beta is an effective and safe treatment for high-risk neuroblastoma, achieving a 54.1% disease control rate and a 5-year overall survival of 63.3%. The manageable safety profile and positive survival outcomes support its use in both first-line and relapsed/refractory settings, providing practical insights for optimizing treatment strategies in pediatric oncology.

## Figures and Tables

**Table 1:** Baseline patient demographics and disease characteristics.

Number of Patients	N = 37
**Patients with HR-NB who received dinutuximab beta, n (%) ^a^**	37 (100)
First line	31 (83.8)
Relapsed disease	6 (16.2)
Distant relapse	4 (10.8)
**Male, n (%)**	26 (70.3)
**Age, median (range) months**	39.2 (0.7–150.4)
**INSS stage at diagnosis, n (%)**	
2	1 (2.7)
3	2 (5.4)
4	27 (73.0)
4S	1 (2.7)
rec ^b^	6 (16.2)
***MYCN* amplified, ^c^ n (%)**	15 (40.5)
INSS stage 2	1 (2.7)
INSS stage 3 ^c^	1 (2.7)
INSS stage 4	11 (29.7)
INSS stage 4S	1 (2.7)
rec ^b^	1 (2.7)
**Unfavorable histopathology, ^d^ n (%)**	30 (81.1)
**Primary tumor major location, n (%)**	
Adrenal glands	34 (91.9)
Abdomen	2 (5.4)
Lymph nodes	1 (2.7)
**Number of metastatic compartments at diagnosis, n (%)**	
0	4 (10.8)
1	11 (29.7)
2	7 (18.9)
3	9 (24.3)
≥4	6 (16.2)

^a^ Refractory disease (misdiagnosis): due to initial misdiagnosis, two patients were incorrectly treated for Wilms tumor and one patient for non-Hodgkin lymphoma, to which there was no response. ^b^ rec: patients received dinutuximab beta as second-line treatment. ^c^ *MYCN* status was not evaluable in one patient with INSS stage 3 disease; this patient received dinutuximab beta in the relapse setting. ^d^ Histopathology was ‘not otherwise specified’ in one patient. CNS, central nervous system; HR-NB, high-risk neuroblastoma; INSS, International Neuroblastoma Staging System.

**Table 2:** Number of dinutuximab beta cycles received and subsequent treatments, including further DB therapy at relapse.

Number of Patients	N = 37
**Number of dinutuximab beta cycles received, n (%)**	
1	4 (10.8)
2	2 (5.4)
3	4 (10.8)
4	2 (5.4)
5	23 (62.2)
6	1 (2.7)
9	1 (2.7)
**Subsequent treatment** **after initial dinutuximab beta course, n (%)**	11 (29.7)
First-line dinutuximab beta	9 (24.3)
RIST	6 (16.2)
MIBG + HSCT	1 (2.7)
Radiotherapy, RIST	1 (2.7)
KIERAN	1 (2.7)
Second-line dinutuximab beta (relapse after 5 first-line cycles)	2 (5.4)
RIST, 1 × N5, 1 × N6, MIBG + haplo-HSCT	1 (2.7)
4 × N5, 4 × N6, MIBG + haplo-HSCT	1 (2.7)
**No further treatment** **after initial dinutuximab beta course, n (%)**	26 (70.3)

The table shows the total number of DB cycles received and treatments administered following the initial DB course, regardless of cycle count. Notably, two patients received additional DB cycles as part of relapse therapy after completing 5 cycles in the first-line setting. These patients are listed under “Second-line dinutuximab beta” in the subsequent treatment section. DB, dinutuximab beta; HSCT, Hematopoietic Stem Cell Transplantation; KIERAN (metronomic therapy), celecoxib, thalidomide, and fenofibrate, with alternating 21-day cycles of low-dose cyclophosphamide and etoposide; MIBG, meta-iodobenzylguanidine; RIST, rapamycin (R) and dasatinib (S) plus chemotherapy with irinotecan (I) and temozolomide (T) [28].

**Table 3:** Treatment response at data cutoff (14 November 2024).

Number of Patients	N = 37 (100%)
**Objective response rate, n (%)**	19 (51.4)
Complete response (CR)	19 (51.4)
Partial response (PR)	0 (0.0)
**Disease control rate, n (%)**	20 (54.1)
Stable disease (SD)	1 (2.7)
**Progressive disease, n (%)**	2 (5.4)
**Death, n (%)**	15 (40.5)

## Data Availability

The original contributions presented in this study are included in the article/Supplementary Materials. Further inquiries can be directed to the corresponding author.

## Supplementary Materials

The following supporting information can be downloaded at: https://www.mdpi.com/article/10.3390/jcm14186641/s1, Table S1: Dinutuximab beta dosing calculations; Table S2: Outcomes for patients treated with dinutuximab in the first-line or the relapsed/refractory setting; Table S3: Grade 3 or 4 adverse events in patients receiving dinutuximab beta; Figure S1: Dinutuximab beta administration; Figure S2: Kaplan–Meier curves for (A) OS and (B) EFS according to the dinutuximab beta regimen (first-line vs. at relapse); and (C) OS and (D) EFS according to *MYCN* amplification status.
