# Supplementary material for: Dinutuximab Beta for the Treatment of High-Risk Neuroblastoma: Data from the Hungarian Pediatric Oncology Network
Source: J Clin Med. 2025 Sep 20;14(18):6641. doi: 10.3390/jcm14186641 (PMC12471212; doi:10.3390/jcm14186641)
Supplement: Supplementary file 1 [file jcm-14-06641-s001.zip › jcm-3831583-supplementary.pdf]

Supplementary file

Table S1. Dinutuximab beta dosing calculations.

Weight	How dosing was calculated
>12 kg	Dosed according to a recent determination of their body surface area, at a rate of 10 mg/m2/day<br>Total dose of dinutuximab beta administered per cycle was 100 mg/m^2 body surface area
<12 kg and >5 kg	Dosed according to their body weight, at a rate of 0.33 mg/kg/day<br>Total dose of dinutuximab beta administered per cycle was 3.3 mg/kg body weight
<5 kg	Dosed according to their body weight, at a rate of 0.22 mg/kg/day<br>Total dose of dinutuximab beta administered per cycle was 2.2 mg/kg body weight

Table S2. Outcomes for patients treated with dinutuximab in the first-line or the relapsed/refractory setting.

Metric	First-line (n=31)	Relapsed/Refractory (n=6)	Source
**Best response — Complete response (CR), n (%)**	16 (51.6)	3 (50.0)	Manuscript text does not provide subgroup breakdown
**Best response — Partial response (PR), n (%)**	—	—	Manuscript text does not provide subgroup breakdown
**Best response — Stable disease (SD), n (%)**	1 (3.2)	—	Manuscript text does not provide subgroup breakdown
**Progressive disease (PD) at cutoff, n (%)**	1 (3.2)	1 (16.6)	Manuscript text does not provide subgroup breakdown
**Deaths at cutoff, n (%)**	13 (41.9)	2 (33.3)	Manuscript text does not provide subgroup breakdown
**Cycle completion — received 5 cycles, n (%)**	19+2* (67.7)	4 (66.6)	Overall: 23/37 received 5 cycles (Suppl. Fig S1)
**Cycle completion — received <5 cycles, n (%)**	10 (32.2)	2* (33.3)	Overall: 12/37 received <5 (reasons in Fig S1)
**Cycle completion — received >5 cycles, n (%)**	—	2* (33.3)	Overall: 2*/37 received >5 cycles (Suppl. Fig S1)
**5-year OS, % (95% CI)**	59% (95% CI 43–80)	83% (95% CI 58–100)	Results section; Suppl. Fig S2
**5-year EFS, % (95% CI)**	55% (95% CI 40–75)	67% (95% CI 38–100)	Results section; Suppl. Fig S2
**Median OS**	Not reached	Not reached	Results section
**Median EFS**	5.1 years (95% CI 2.5–NA)	Not reached	Results section

CI, confidence interval; EFS, event-free survival; OS, overall survival.

*One patient received 6 cycles of dinutuximab beta in total, 5 cycles first-line and an additional cycle to treat relapsed disease (RIST [rapamycin (R) and dasatinib (S) plus chemotherapy with irinotecan (I) and temozolomide (T)] [28], 1 cycle of N5, 1 cycle of N6, and MIBG plus haploidentical hematopoietic stem cell transplant [haplo-SCT]); therapy was discontinued due to the patient's death. One further patient received 9 cycles of dinutuximab beta, 5 cycles first-line (SIOPEN HR-NBL) and an additional 4 cycles to treat relapsed disease (4 cycles of N5, 4 cycles of N6, and MIBG + haplo-SCT).

Table S3. Grade 3 or 4[a] adverse events in patients receiving dinutuximab beta.

**Number of patients**	**N=37**
**CTCAE term, n (%)**	
Blood and lymphatic system disorders – other	14 (37.8)
Hypoxia	14 (37.8)
Hepatobiliary disorders – other[b]	11 (29.2)
Hypotension	10 (27.0)
Capillary leak syndrome[c]	5 (13.5)
Diarrhea	3 (8.1)
Generalized edema	2 (5.4)
Urinary tract infection	2 (5.4)
Acute respiratory distress syndrome[c]	1 (2.7)
Allergic disorders	1 (2.7)
Anaphylaxis	1 (2.7)
Depressed level of consciousness	1 (2.7)
Device related infection	1 (2.7)
EBV reactivation	1 (2.7)
Fever	1 (2.7)
Gastrointestinal infection	1 (2.7)
Herpes simplex	1 (2.7)
Pulmonary infection	1 (2.7)
Pulmonary edema	1 (2.7)
Skin infection	1 (2.7)
Typhlitis[c]	1 (2.7)

[a]Only five (13.5%) patients had Grade 4 adverse events. [b]Grade 4 events in two (5.4%) patients. [c]Grade 4 event in one (2.7%) patient.CTCAE, Common Terminology Criteria for Adverse Events; EBV, Epstein-Barr virus.

Figure S1. Dinutuximab beta administration.

Patients included (N=37)

Patients receiving 5 cycles (n=23)

Patients receiving <5 cycles (n=12) due to:

- Death (n=7)
- Severe neurological side effects (n=1)
- Acute respiratory distress syndrome (n=1)
- Still undergoing therapy at the time of data collection cut-off (n=2)
- Only receiving a permit for 3 cycles of dinutuximab beta from the National Health Insurance Fund of Hungary (n=1)

Patients receiving >5 cycles (n=2):

- One patient received 6 cycles of dinutuximab beta in total; 5 cycles first-line and an additional cycle to treat relapsed disease; therapy was discontinued due to the patient's death
- One patient received 9 cycles of dinutuximab beta; 5 cycles first-line and an additional 4 cycles to treat relapsed disease

Figure S2. Kaplan–Meier curves for (**A**) OS and (**B**) EFS according to the dinutuximab beta regimen (first-line vs. at relapse); and (**C**) OS and (D) EFS according to *MYCN* amplification status. Event timelines were calculated from initial diagnosis. Note: Two patients received dinutuximab beta both as part of first-line maintenance and relapse therapy; for the purpose of this analysis, these patients were included in the first-line group, reflecting the initial treatment intent. EFS, event-free survival; OS, overall survival.

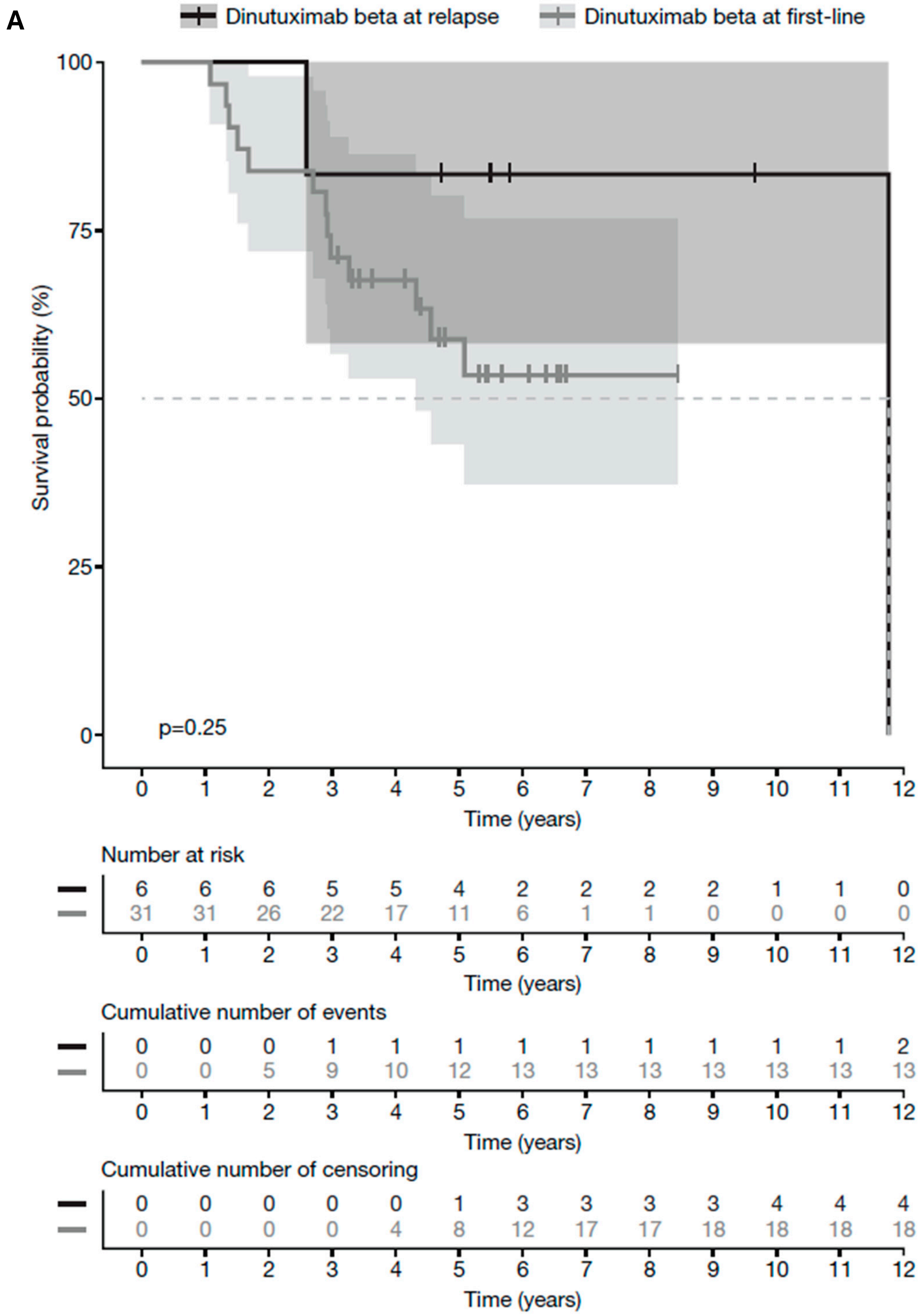

B

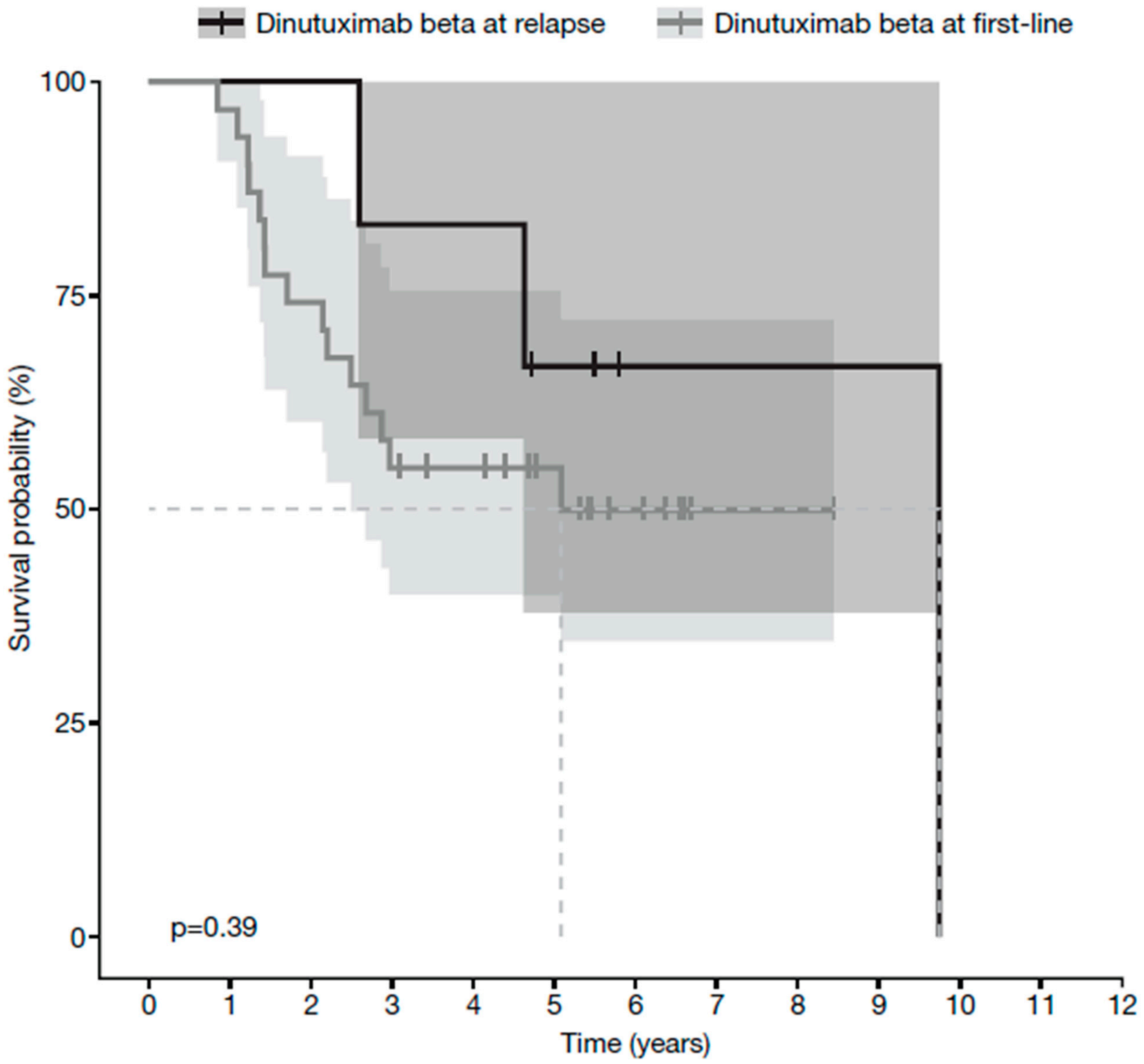

Number at risk

	0	1	2	3	4	5	6	7	8	9	10	11	12
Relapse	6	6	6	5	5	3	1	1	1	1	0	0	0
First-line	31	30	23	17	15	11	6	1	1	0	0	0	0

Time (years)

Cumulative number of events

	0	1	2	3	4	5	6	7	8	9	10	11	12
Relapse	0	0	0	1	1	2	2	2	2	2	3	3	3
First-line	0	1	8	14	14	14	15	15	15	15	15	15	15

Time (years)

Cumulative number of censoring

	0	1	2	3	4	5	6	7	8	9	10	11	12
Relapse	0	0	0	0	0	1	3	3	3	3	3	3	3
First-line	0	0	0	0	2	6	10	15	15	16	16	16	16

Time (years)

C

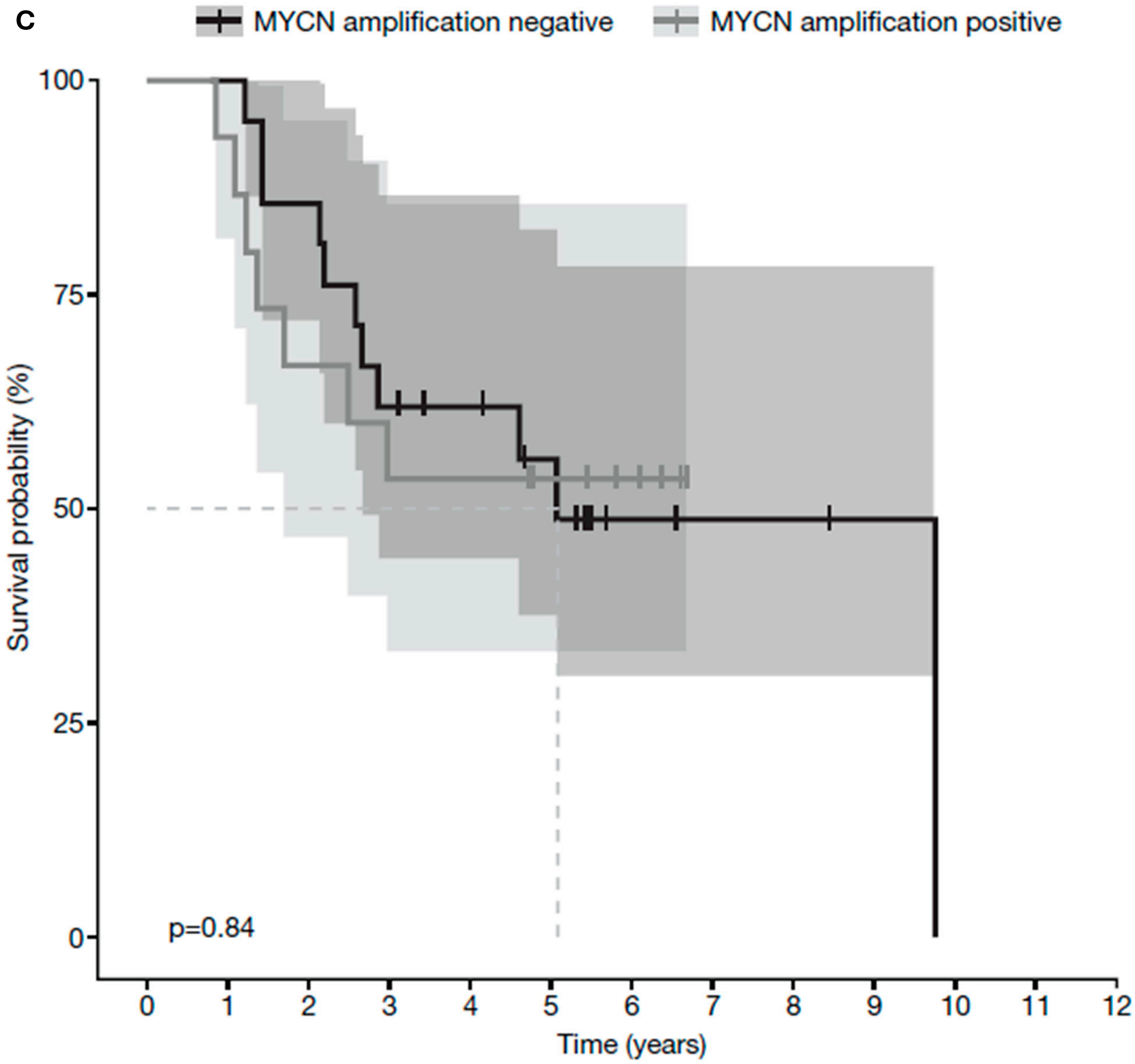

Number at risk

	0	1	2	3	4	5	6	7	8	9	10	11	12
MYCN amplification negative	21	21	18	13	11	8	3	2	2	1	0	0	0
MYCN amplification positive	15	14	10	8	8	6	4	0	0	0	0	0	0

Time (years)

Cumulative number of events

	0	1	2	3	4	5	6	7	8	9	10	11	12
MYCN amplification negative	0	0	3	8	8	9	10	10	10	10	11	11	11
MYCN amplification positive	0	1	5	7	7	7	7	7	7	7	7	7	7

Time (years)

Cumulative number of censoring

	0	1	2	3	4	5	6	7	8	9	10	11	12
MYCN amplification negative	0	0	0	0	2	4	8	9	9	10	10	10	10
MYCN amplification positive	0	0	0	0	0	2	4	8	8	8	8	8	8

Time (years)

D

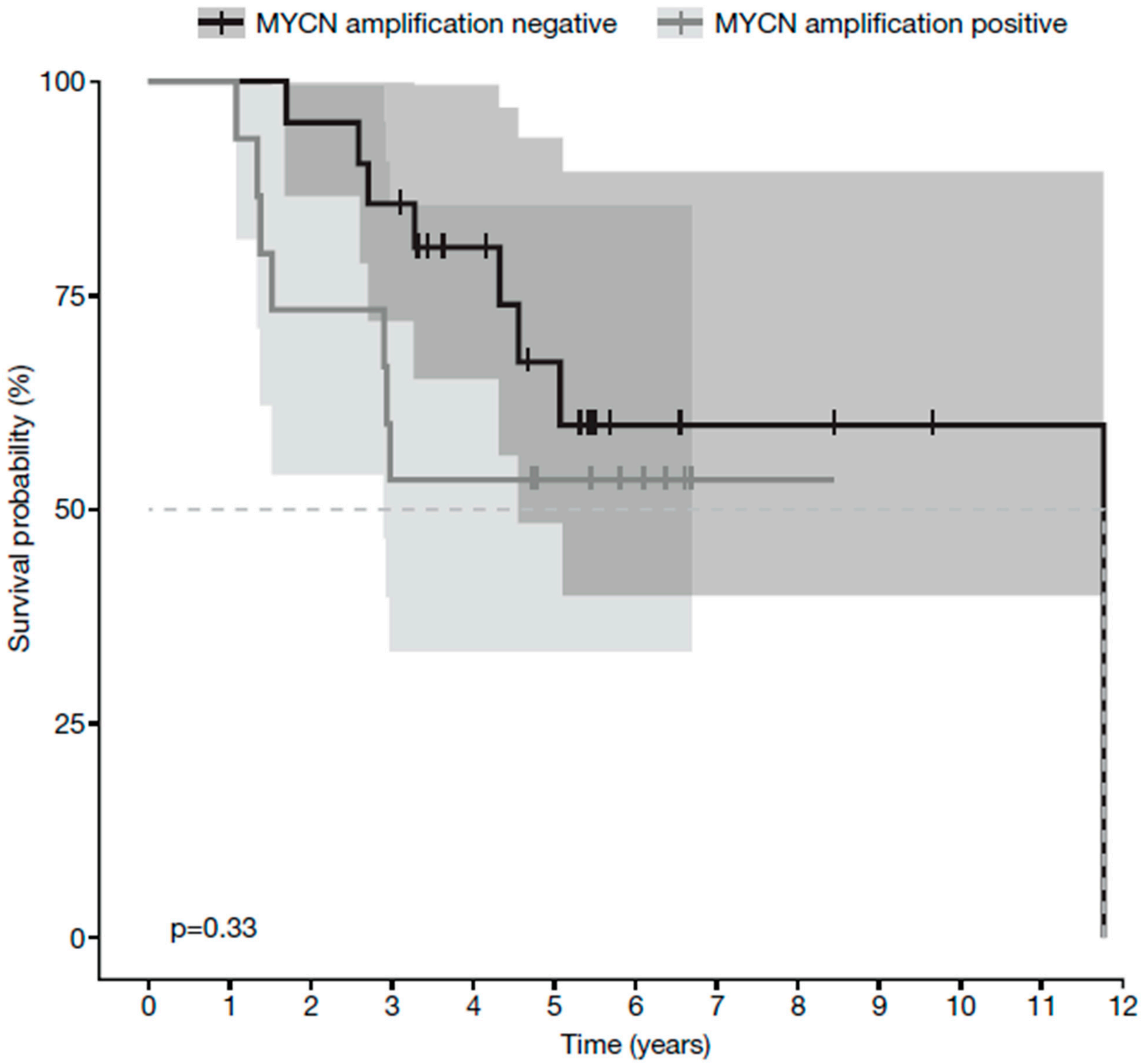

Number at risk

	0	1	2	3	4	5	6	7	8	9	10	11	12
MYCN amplification negative (black)	21	21	20	18	13	9	4	3	3	2	1	1	0
MYCN amplification positive (grey)	15	15	11	8	8	6	4	0	0	0	0	0	0

Time (years)

Cumulative number of events

	0	1	2	3	4	5	6	7	8	9	10	11	12
MYCN amplification negative (black)	0	0	1	3	4	6	7	7	7	7	7	7	8
MYCN amplification positive (grey)	0	0	4	7	7	7	7	7	7	7	7	7	7

Time (years)

Cumulative number of censoring

	0	1	2	3	4	5	6	7	8	9	10	11	12
MYCN amplification negative (black)	0	0	0	0	4	6	10	11	11	12	13	13	13
MYCN amplification positive (grey)	0	0	0	0	0	2	4	8	8	8	8	8	8

Time (years)
